# Zinc Prevents Sickness Behavior Induced by Lipopolysaccharides after a Stress Challenge in Rats

**DOI:** 10.1371/journal.pone.0120263

**Published:** 2015-03-16

**Authors:** Thiago B. Kirsten, Marcella C. Galvão, Thiago M. Reis-Silva, Nicolle Queiroz-Hazarbassanov, Maria M. Bernardi

**Affiliations:** 1 Department of Pathology, School of Veterinary Medicine, University of São Paulo, São Paulo, Brazil; 2 Environmental and Experimental Pathology, Paulista University, São Paulo, Brazil; Brock University, CANADA

## Abstract

Sickness behavior is considered part of the specific beneficial adaptive behavioral and neuroimmune changes that occur in individuals in response to infectious/inflammatory processes. However, in dangerous and stressful situations, sickness behavior should be momentarily abrogated to prioritize survival behaviors, such as fight or flight. Taking this assumption into account, we experimentally induced sickness behavior in rats using lipopolysaccharides (LPS), an endotoxin that mimics infection by gram-negative bacteria, and then exposed these rats to a restraint stress challenge. Zinc has been shown to play a regulatory role in the immune and nervous systems. Therefore, the objective of this study was to examine the effects of zinc treatment on the sickness response of stress-challenged rats. We evaluated 22-kHz ultrasonic vocalizations, open-field behavior, tumor necrosis factor α (TNF-α), corticosterone, and brain-derived neurotrophic factor (BDNF) plasma levels. LPS administration induced sickness behavior in rats compared to controls, i.e., decreases in the distance traveled, average velocity, rearing frequency, self-grooming, and number of vocalizations, as well as an increase in the plasma levels of TNF-α, compared with controls after a stressor challenge. LPS also decreased BDNF expression but did not influence anxiety parameters. Zinc treatment was able to prevent sickness behavior in LPS-exposed rats after the stress challenge, restoring exploratory/motor behaviors, communication, and TNF-α levels similar to those of the control group. Thus, zinc treatment appears to be beneficial for sick animals when they are facing risky/stressful situations.

## Introduction

The concept that pathogens, infections and inflammatory processes induce sickness in a specific way in several species was documented more than 25 years ago by Hart [1]. Animals usually present common symptoms even when affected by different infectious processes, such as bacteria, viruses and fungi [1–3]. These behavioral and neuroimmune responses were named ‘sickness behavior’ by Kent and colleagues [4]. Sickness behavior is generally accompanied by fever; prostration; decreases in in exploratory activity, social behavior, feeding behavior, and sexual behavior; and the induction of anhedonia, as well as poor learning and cognitive functions [2, 5].

Sickness behavior is normally a temporary state characterized by adaptive behavioral- and neuroimmune-specific changes orchestrated by the host to fight the invading microorganism and heal more quickly, as well as reduce exposure of the sick animal to predation and contamination of their colony [1, 4]. However, sickness behavior is considered a motivational state that can be modulated by the environmental context in which the animal finds itself [6]. In other words, in situations where the animal is at risk of death or engaged in a hierarchical confrontation (e.g., with predators, competitors, and climatic extremes), sickness behavior is momentarily interrupted to prioritize other behaviors, such as fight or flight and maternal behavior [6, 7]. Thus, in stressful situations, it is better to prioritize the so-called fight or flight response than sickness behavior.

Lipopolysaccharides (LPS) are an endotoxin that mimics infection by gram-negative bacteria by activating the immune system to release proinflammatory cytokines, such as tumor necrosis factor α (TNF-α), interleukin-1β (IL-1β), and IL-6 [8, 9]. LPS also activates the hypothalamic-pituitary-adrenal (HPA) axis [10]. LPS is considered a potent sickness behavior inducer [11].

Zinc is known to play a regulatory role in the immune and nervous systems, participating in innate and adaptive immunity [12, 13]. Thus, zinc is currently recommended for the treatment of many illnesses, including flu, respiratory infections, and pneumonia [13, 14]; however, there are usually no concerns about stressful intercurrences. Considering that sickness behavior can be differentially expressed during stressful situations, we experimentally induced sickness behavior in rats via the administration of LPS and subsequently exposed these rats to a restraint stress challenge. The objective of this study was to verify the effects of zinc treatment on the sickness response of stress-challenged rats.

We administered LPS to rats, followed by zinc, then exposed them to a restraint stress. We evaluated 22-kHz ultrasonic vocalizations (emitted in aversive contexts [15]), open-field behavior (to evaluate exploratory/motor and anxiety parameters [16, 17]), and plasma TNF-α, corticosterone (an indicator of stress and HPA axis activity [18]), and levels of brain-derived neurotrophic factor (BDNF, as LPS and zinc may interfere with BDNF expression [19, 20]). To the best of our knowledge, this is the first study evaluating zinc supplementation, sickness behavior, and stress.

## Materials and Methods

### Ethics statement

This study was performed in strict accordance with the recommendations of the Guide for the Care and Use of Laboratory Animals by the National Institutes of Health. The protocol was approved by the Committee on the Ethics of Animal Experiments of the School of Veterinary Medicine, University of São Paulo, Brazil (permit no. 3130/2013), a committee whose guidelines are based on the guidelines of the National Institutes of Health. All efforts were made to minimize suffering. The experiments were performed in accordance with good laboratory practice and quality assurance methods.

### Animals, treatments and experimental design

A total of 40 male Wistar rats, 12 weeks of age, were used. They were housed in polypropylene cages (38 X 32 X 16 cm; 3–4 rats per cage) at a controlled temperature (22°C ± 2°C) and humidity (65–70%) with artificial lighting (12-hr light/12-hr dark cycle, lights on at 6:00 AM). The animals had free access to Nuvilab rodent chow (Nuvital Co., Sao Paulo, Brazil) and filtered water. Sterilized and residue-free wood shavings were used for animal bedding. All of the experiments, including treatments and behavioral observations, were performed between 8:00 AM and 1:00 PM to minimize the effects of circadian rhythms.

The rats were randomly divided into four groups (n = 10 per group). (1) SAL+SAL, rats that received sterile saline (0.9% NaCl, 0.2 ml/100 g, intraperitoneally [i.p.]) and then one hour later, received an additional saline injection (0.2 ml/100 g, subcutaneously [s.c.] in the nape of the neck). The SAL+SAL group was also referred to as the control group. Saline served as the vehicle for both LPS and zinc. (2) LPS+SAL, rats that received LPS solution (from *Escherichia coli*; Sigma, St. Louis, MO; serotype 0127: B8, in sterile saline 50 μg/ml and administered at a dose of 100 μg/kg, i.p.) and then one hour later, received a saline injection (0.2 ml/100 g, s.c.). The LPS dose and time interval were chosen because they were reported to cause sickness behavior and proinflammatory cytokine and glucocorticoid release following LPS administration [21–23]. (3) LPS+Zn, rats that received LPS solution (100 μg/kg, i.p.) and then one hour later, received zinc (zinc sulfate heptahydrate, ZnSO_4_, Sigma, St. Louis, MO, USA, cat. no. Z0635; 2 mg/kg in saline, s.c.). The zinc dose was chosen based on the studies of Chua and colleagues [24]. A subcutaneous zinc injection induces an immediate and reproducible increase in plasma zinc levels that peak at levels 4- to 5-fold higher than normal 2 h after injection and return to normal within 12 h [25]. (4) SAL+Zn, rats that received saline (0.2 ml/100 g, i.p.) and then one hour later, received zinc (ZnSO_4_, 2 mg/kg, s.c.). One hour after the second injection, each rat from each group was placed individually in restraint tubes for a 2-hour session and then evaluated for behavioral and neuroimmune parameters. Thus, the interval between the first injection and the end of the restraint stress was 4 hours.

### Restraint stress

Restraint stress is considered a simple and painless model of stress that does not cause any lasting impairment [26]. It is considered a model of psychological stress due to its similarity to the natural experience of confinement [27]. The restraint stress apparatus consisted of plastic cylindrical restraint tubes (6.5 cm diameter, 20 cm length) for individual restraints that were fixed on a table with both ends closed and holes for the tail and ventilation. The design of the tubes prevented pain and compression. The rats were subjected to a single restraint session of 2 hours. A 2-hour session is considered sufficient to activate the HPA axis, increasing circulating corticosterone levels [26, 28].

### Ultrasonic vocalization

During the final 5 min of restraint (i.e., after 115 min of restraint), the rats were observed for 22-kHz ultrasonic vocalizations within the restraint tube. The vocalization test room was acoustically isolated. It is known that 22-kHz ultrasonic vocalizations are emitted in aversive contexts, such as in the presence of a predator and footshock cues [15, 29]. Ultrasonic vocalizations were detected using Ultravox software (Noldus Information Technology, Leesburg, VA, USA) with a filter and ultrasonic microphone that was tuned to a range centered at 22 kHz and placed 1 cm away from the restraint tube. Several parameters were automatically recorded during the 5-min session, including the number of vocalizations, total vocalization time, mean vocalization duration, maximal vocalization duration, minimal vocalization duration, total silence duration, mean silence duration interval, maximal silence duration interval, and minimal silence duration interval. The durations were recorded in seconds.

### Open-field behavior

Immediately after the ultrasonic vocalization test, the rats were removed from the restraint tube and observed in an open field to evaluate exploratory/motor and anxiety behaviors [16, 17]. The open-field apparatus consisted of a round wooden arena (90 cm diameter, 28 cm high walls) that was painted gray with an acrylic washable cover. The testing room was a small room with dim lighting. Each rat was individually placed in the center of the apparatus, and the following parameters were automatically or manually recorded using EthoVision software (Noldus Information Technology, Leesburg, VA, USA) over a period of 5 min: traveled distance (cm), average velocity (cm/s), rearing frequency, self-grooming (s), and time spent in the central and peripheral zones (s). A video camera mounted 100 cm above the arena was used to collect the data, which were analyzed using EthoVision software installed on a compatible computer placed in an adjacent room. The apparatus was washed with a 5% alcohol/water solution before placement of the animals to obviate possible biasing effects from odor cues left by previous rats.

### TNF-α, corticosterone and BDNF

Following the open-field test, the rats were decapitated, and trunk blood was collected in conical tubes that contained 10% ethylenediaminetetraacetic acid (EDTA). The samples were centrifuged, and plasma was obtained. Plasma samples of each animal were aliquoted in several conical tubes for separate analyses of TNF-α, corticosterone, and BDNF using enzyme-linked immunosorbent (ELISA) commercial kits in duplicate and according to the manufacturer’s instructions.

TNF-α is produced and released after the administration of LPS and is a biomarker of sickness behavior [3, 11, 30]. Corticosterone is the most abundant circulating steroid secreted by rodents and is considered to be a good indicator of HPA axis activity in these species [18]. BDNF is a neurotrophin that is found throughout the brain, central nervous system, and peripheral blood. It regulates neuronal survival, morphology, development, and function and plays a critical role in synaptogenesis and synaptic plasticity [31]. Furthermore, previous studies have shown that LPS and zinc interfere with BDNF expression [19, 20, 32, 33].

TNF-α was quantified using the DuoSet R&D Systems kit (cat. no. DY510, Minneapolis, MN, USA). The results are expressed in pg/ml. Corticosterone levels were determined using an Arbor Assays kit (cat. no. K014-H, Ann Arbor, MI, USA). The results are expressed in ng/ml. BDNF levels were determined using a Promega kit (cat. no. G7610, Madison, WI, USA). The results are expressed in pg/ml. We evaluated the levels of free mature BDNF (i.e., non-acidified samples) and those of total free BDNF (i.e., acid-treated and neutralized samples, which indicate the pro-form of BDNF and mature BDNF, respectively). Mature BDNF is the active form of this neurotrophin, which binds to the TrkB receptor (i.e., neurotrophin receptors) [34].

### Statistical analysis

Homogeneity was verified using Bartlett’s test. Normality was verified using the Kolmogorov-Smirnov test. One-way analysis of variance (ANOVA) followed by Tukey’s multiple-comparison test was used to compare parametric data. The results are expressed as the mean ± SEM. In all cases, the results were considered significant at *p* < 0.05.

## Results

**Fig 1:**
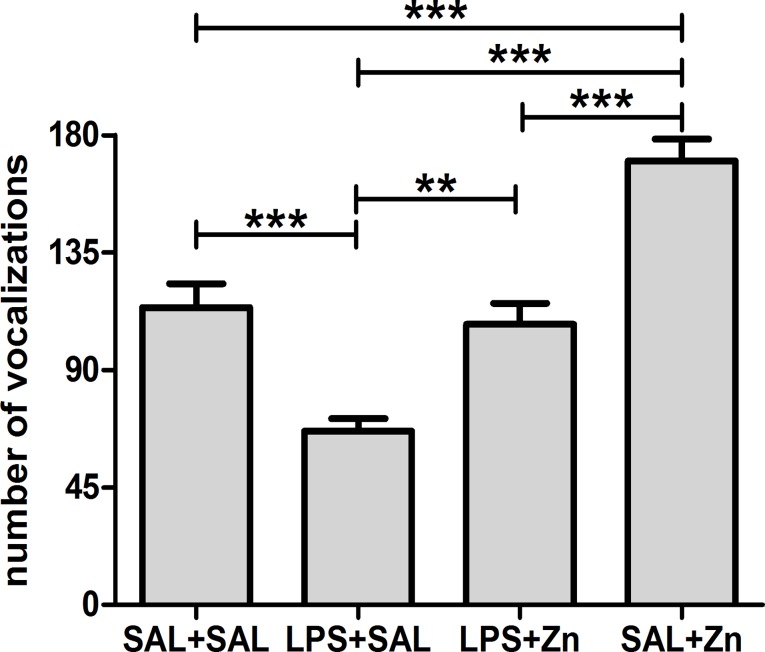
Number of vocalizations. Effects of LPS (100 μg/kg) and zinc (ZnSO_4_; 2 mg/kg) on the number of 22-kHz ultrasonic vocalizations in adult male rats after a restraint stress challenge. SAL+SAL, saline injection followed by another saline injection 1 h later; LPS+SAL, LPS injection followed by a saline injection 1 h later; LPS+Zn, LPS injection followed by a zinc injection 1 h later; SAL+Zn, saline injection followed by a zinc injection 1 h later (*n* = 10 per group). **p < 0.01, ***p < 0.0001 (one-way ANOVA followed by the Tukey test). The data are expressed as the mean ± SEM.

One-way ANOVA demonstrated that the number of 22-kHz vocalizations after a stressor challenge was influenced by LPS and zinc administration (p<0.0001, Fig. 1). The multiple comparisons test revealed that LPS administration (LPS+SAL group) decreased the number of vocalizations compared with those of the control group (SAL+SAL). Treatment with zinc after LPS (LPS+Zn group) prevented the reduction in vocalizations, restoring vocalizations to the levels found in the control group. The administration of zinc without LPS (SAL+Zn group) increased the number of vocalizations compared with those of the other three groups. The other parameters associated with ultrasonic vocalizations were found to be similar among the four groups (S1 Table).

**Fig 2:**
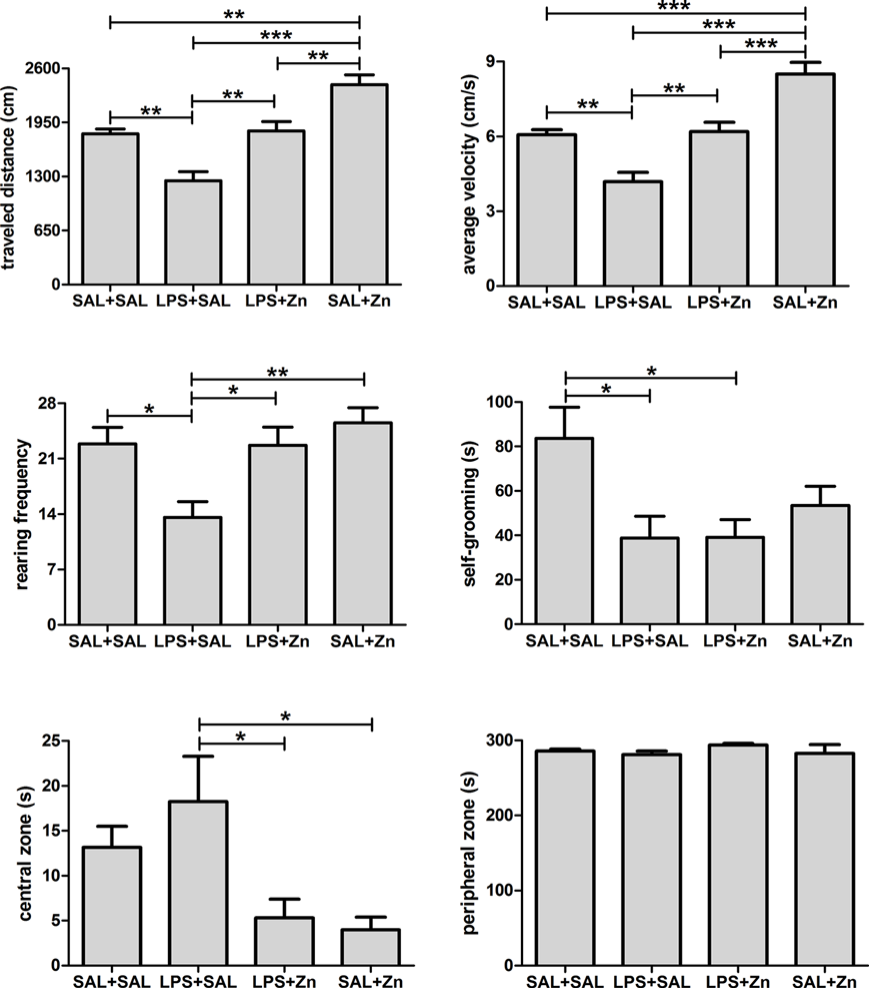
Open-field behavior. Effects of LPS (100 μg/kg) and zinc (ZnSO_4_; 2 mg/kg) on the open-field behaviors in adult male rats after a restraint stress challenge. SAL+SAL, saline injection followed by another saline injection 1 h later; LPS+SAL, LPS injection followed by a saline injection 1 h later; LPS+Zn, LPS injection followed by a zinc injection 1 h later; SAL+Zn, saline injection followed by a zinc injection 1 h later (*n* = 10 per group). *p < 0.05, **p < 0.01, ***p < 0.0001 (one-way ANOVA followed by the Tukey test). The data are expressed as the mean ± SEM.

One-way ANOVA demonstrated that the general open-field activity of rats after a stressor challenge was influenced by LPS and zinc administration (Fig. 2). There were differences in the traveled distance and average velocity (p<0.0001 for both parameters). The multiple comparisons test revealed that LPS administration (LPS+SAL group) decreased the traveled distance and the average velocity of rats compared to those of the control group (SAL+SAL). Treatment with zinc after LPS (LPS+Zn group) prevented the reduction in the traveled distance and average velocity, restoring these outcomes to the same levels found in the control group. The administration of zinc without LPS (SAL+Zn group) increased the traveled distance and average velocity of the rats compared with those of the other three groups. There were also differences in the rearing frequency (p = 0.0014). The multiple comparisons test revealed that LPS administration (LPS+SAL group) decreased the rearing frequency of rats compared with that of the control group (SAL+SAL). Treatment with zinc after LPS (LPS+Zn group) prevented the reduction in rearing frequency, restoring the frequency to the level found in the control group. The rearing frequency was found to be similar when the SAL+Zn group was compared with the SAL+SAL and LPS+Zn groups. In addition, there were differences in the duration of self-grooming (p = 0.0130). The multiple comparisons test revealed that LPS administration (LPS+SAL group) decreased the self-grooming of rats compared with that of the control group (SAL+SAL). Zinc treatment with or without LPS (the LPS+Zn and SAL+Zn groups) was unable to prevent the reduction in self-grooming, exhibiting similar values to those in the LPS+SAL group. There were also differences in the amount of time spent in the central zone (p = 0.0062). The multiple comparisons test revealed that zinc treatment (the LPS+Zn and SAL+Zn groups) reduced the amount of time spent in the central zone compared with that of rats in the LPS+SAL group; however, no differences were observed between the zinc treatment groups (LPS+Zn and SAL+Zn) and the SAL+SAL group. Finally, there were no differences in the amount of time spent in the peripheral zone among the four groups (p = 0.5257).

**Fig 3:**
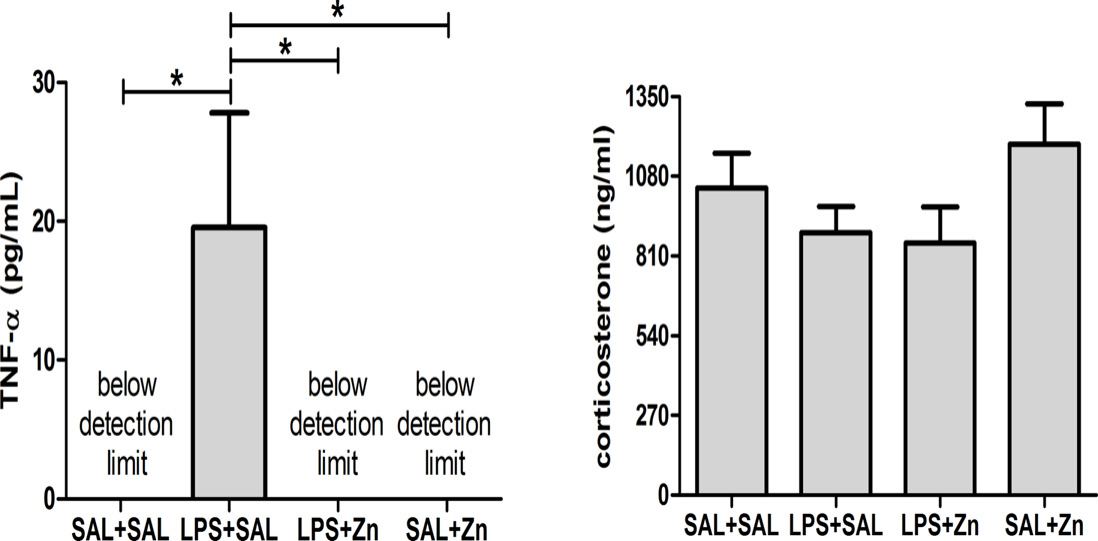
TNF-α and corticosterone. Effects of LPS (100 μg/kg) and zinc (ZnSO_4_; 2 mg/kg) on TNF-α and corticosterone plasma levels in adult male rats after a restraint stress challenge. SAL+SAL, saline injection followed by another saline injection 1 h later; LPS+SAL, LPS injection followed by a saline injection 1 h later; LPS+Zn, LPS injection followed by a zinc injection 1 h later; SAL+Zn, saline injection followed by a zinc injection 1 h later (*n* = 10 per group). *p < 0.05 (one-way ANOVA followed by the Tukey test). The data are expressed as the mean ± SEM.

One-way ANOVA revealed that the TNF-α plasma levels of rats were influenced by the administration of LPS after a stressor challenge (p = 0.0031, Fig. 3). The multiple comparisons test revealed that the administration of LPS (LPS+SAL group) increased TNF-α levels compared with those of the control group (SAL+SAL). As expected, there were no detectable levels of TNF-α in the rats that were not treated with LPS (the SAL+SAL and SAL+Zn groups). Interestingly, treatment with zinc after LPS (LPS+Zn group) prevented the release of TNF-α into the plasma, resulting in levels that were similar to those of the control group. There were no differences in corticosterone plasma levels among the four groups (p = 0.1831, Fig. 3). Thus, HPA axis activity does not seem to be differentially affected by LPS and zinc treatment.

**Fig 4:**
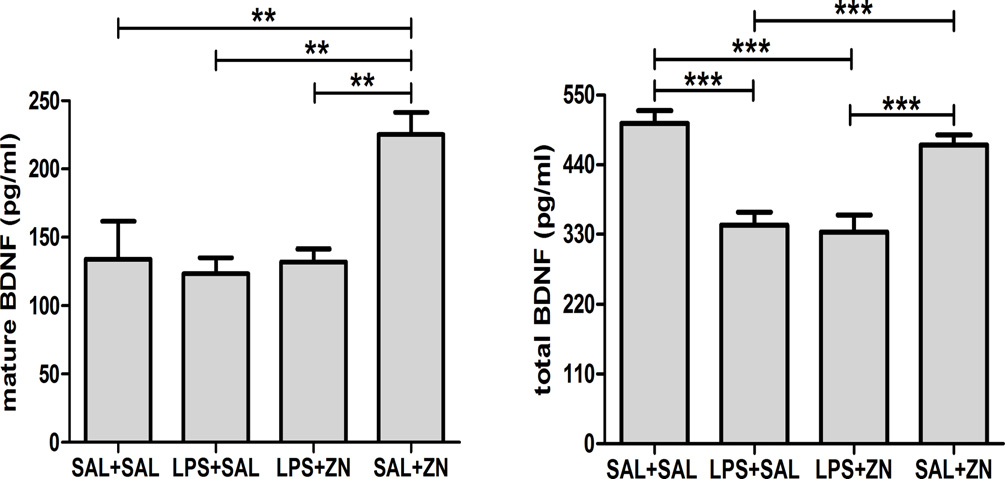
BDNF. Effects of LPS (100 μg/kg) and zinc (ZnSO_4_; 2 mg/kg) on mature and total BDNF plasma levels in adult male rats after a restraint stress challenge. SAL+SAL, saline injection followed by another saline injection 1 h later; LPS+SAL, LPS injection followed by a saline injection 1 h later; LPS+Zn, LPS injection followed by a zinc injection 1 h later; SAL+Zn, saline injection followed by a zinc injection 1 h later (*n* = 10 per group). **p < 0.01, ***p < 0.0001 (one-way ANOVA followed by the Tukey test). The data are expressed as the mean ± SEM.

One-way ANOVA revealed that the plasma levels of BDNF in the rats after a stressor challenge were influenced by LPS and zinc administration (Fig. 4). There were observable differences in the mature BDNF levels (p = 0.0006). The multiple comparisons test revealed that zinc administration (SAL+Zn group) increased the levels of mature BDNF compared with those of the other three groups. There were also differences observed in the total BDNF levels (p<0.0001). The multiple comparisons test revealed that LPS administration (the LPS+SAL and LPS+Zn groups) decreased the total levels of BDNF compared with those of the control group (SAL+SAL). The administration of zinc without LPS (SAL+Zn group) resulted in levels that were similar to those of the control group.

## Discussion

LPS administration (the LPS+SAL group) decreased traveled distance, average velocity, rearing frequency, and self-grooming in rats compared with controls. In other words, LPS induced prostration and decreased exploratory activity, which are typical symptoms of sickness behavior [2, 35]. LPS also increased TNF-α plasma levels, another typical sign of the sickness behavior response [4, 30]. Thus, rats presented the main behavioral and immune changes associated with sickness behavior four hours after LPS administration (100 μg/kg, i.p), even after a stressor challenge.

Rodents emit ultrasonic vocalizations in a variety of situations [36]. Rats use ultrasonic vocalizations for communication in aversive contexts (i.e., the presence of a predator, shocks) and pleasant contexts (i.e., play behavior, food cues, copulation) [15]. For example, if a dominant male rat behaves aggressively toward a submissive rat, the submissive rat emits 22-kHz ultrasonic vocalizations to communicate with the dominant rat to reduce his aggressive behavior [37]. Other contexts, such as feeding and ambulation, also elicit specific ultrasonic vocalizations [15]. Thus, ultrasonic vocalization is a complex and important communication tool for rodents that carries both emotional and environmental information.

The number of vocalizations was reduced in response to LPS administration. To the best of our knowledge, the present study is the first to report changes in ultrasonic vocalization during sickness behavior. In addition to prostration, decreased exploratory activity, and increased TNF-α levels, sickness behavior was accompanied by a reduction in ultrasonic vocalizations. This finding is in accordance with findings from the literature that report a decrease in social behavior during sickness behavior [2, 38]. This communication impairment may represent an adaptive response for the species because it may reduce the likelihood that the sick animal will contaminate its colony [1]. Because these interesting findings regarding changes in ultrasonic vocalization agree with the classical changes associated with sickness behavior, we suggest using the ultrasonic vocalization test in other studies involving sickness behavior analysis. The ultrasonic vocalization test could be used in a complementary manner with other classical parameter evaluations.

Zinc treatment increased the travel distance, average velocity, rearing frequency, and number of vocalizations after LPS exposure (i.e., the LPS+Zn group versus the LPS+SAL group), restoring the same values observed in the control group. Zinc also prevented TNF-α plasma release after LPS exposure. Thus, zinc induced behavioral and immune changes, preventing the expression of sickness behavior in LPS-exposed rats after a stressor challenge.

In stressful situations, such as life-threatening events, it is important and beneficial to momentarily interrupt sickness behavior to prioritize behaviors related to survival, such as fight or flight [6]. Thereby, zinc treatment after LPS exposure allowed a beneficial adaptation in rats facing a stressful situation by allowing them to interrupt sickness behavior, returning their behavior and immune system to levels similar to those of the control group. In the context of the restraint stress challenge, it was beneficial to momentarily interrupt sickness behavior to address “the problem.” Zinc allowed the animals to interrupt sickness behavior during stress, most likely increasing their chances of survival.

Zinc is one of the most important trace elements in mammals, and it is required for many physiological processes, such as cell proliferation and differentiation, growth and development, and the regulation of enzymatic activity and innate and adaptive immunity [13, 14, 39]. It is likely that zinc treatment is beneficial after LPS exposure because cytokines, which are produced after LPS exposure, induce metallothionein, which sequesters zinc and induces hypozincemia [40, 41]. Subcutaneous zinc injection or dietary zinc supplementation prevents LPS-induced hypozincemia and reproductive and offspring behavioral impairments in mice [40–42].

However, zinc treatment in rats that are not infected/inflamed is not recommended because the rats in the SAL+Zn group exhibited increases in the number of vocalizations, traveled distance and average velocity compared with those of the control group. Thus, rats of the SAL+Zn group presented abnormal behaviors, including increases in communication and hyperlocomotion.

The open field test is a popular animal test for evaluating anxiety-like behavior. As reported in a review by Prut and Belzung [17], rodents spontaneously prefer spending time in the periphery of the apparatus compared with the central parts of the open field. Mice and rats walk along the walls, a behavior called thigmotaxis. An increase in the time spent in the central portion of the field indicates anxiolysis, whereas a decrease in time spent in the central part of the device can be interpreted as an anxiogenic effect. Thus, the fact that the zinc treatment (LPS+Zn and SAL+Zn groups) decreased the amount of time spent within the central zone, compared with that of the LPS+SAL group, together with decreased self-grooming suggests that zinc induced an anxiogenic effect. However, we cannot definitively conclude that zinc has an anxiogenic effect because (1) there was no change in the time spent in the peripheral zone (anxiogenic rats tend to spend more time in the periphery of the apparatus); (2) the zinc treatment induced hyperlocomotion, which can affect the anxiety analysis; and (3) the zinc treatment (LPS+Zn and SAL+Zn groups) had no effect on the time spent in the central zone compared with that observed in the control group. Thus, zinc influenced motor/exploratory behaviors and did not significantly affect anxiety-like behaviors.

One of the mechanisms of the neuroinflammation produced by LPS exposure may be the interference with BDNF gene expression and function [19]. Injection of LPS has been reported to significantly decrease BDNF expression of several brain regions [43]. Cytokines, such as IL-1β, released during the immune response to LPS impair BDNF induction in the rat hippocampus [19, 44]. Our results confirmed that the administration of LPS decreased total BDNF expression in the blood plasma. Our report of TNF-α induction after LPS exposure also confirmed that the mechanism underlying the decrease in BDNF levels after LPS exposure is associated with cytokine induction. Particularly, we demonstrated the relevance of TNF-α to BDNF interference.

Zinc treatment was unable to prevent the reduction in total BDNF levels after the administration of LPS. Interestingly, previous studies have shown that zinc supplementation, either by injections [20] or dietary sources [33], may induce BDNF expression. Based in our data, we speculate that zinc should have activated more BDNF mature forms, compared with those of the control group. It is possible that the zinc dose used in the present study was not sufficient to affect total BDNF expression after LPS exposure, as the other studies administered higher doses of zinc [20, 33]. However, based on our findings of abnormal behavior, such as hyperlocomotion, we do not recommend higher doses of zinc for clinical purposes. We also would not recommend higher doses of zinc because zinc administration without LPS exposure increased both plasma mature and total BDNF levels. Furthermore, zinc was found to have negative consequences for spatial memory [45].

In conclusion, LPS administration induced sickness behavior in rats, even after a stressor challenge. Sickness behavior is considered a beneficial behavioral strategy in response to infectious/inflammatory processes. However, in dangerous and stressful situations, sickness behavior should be momentarily abrogated. During these stressful situations, it is better to prioritize survival behaviors, such as fight or flight. Zinc treatment was able to prevent sickness behavior in LPS-exposed rats after the stress challenge. Therefore, zinc treatment allowed the sick animal to respond more appropriately to risky situations.

## Supporting Information

S1 Table Ultrasonic vocalizations. Effects of LPS (100 μg/kg) and zinc (ZnSO_4_; 2 mg/kg) on the 22-kHz ultrasonic vocalizations in adult male rats after a restraint stress challenge. SAL+SAL, saline injection followed by another saline injection 1 h later; LPS+SAL, LPS injection followed by a saline injection 1 h later; LPS+Zn, LPS injection followed by a zinc injection 1 h later; SAL+Zn, saline injection followed by a zinc injection 1 h later (*n* = 10 per group). The data are expressed as the mean ± SEM.
(DOCX)
