# Supplementary material for: Zinc Prevents Sickness Behavior Induced by Lipopolysaccharides after a Stress Challenge in Rats
Source: PLoS One. 2015 Mar 16;10(3):e0120263. doi: 10.1371/journal.pone.0120263 (PMC4361539; doi:10.1371/journal.pone.0120263)
Supplement: S1 Table — Effects of LPS (100 μg/kg) and zinc (ZnSO4; 2 mg/kg) on the 22-kHz ultrasonic vocalizations in adult male rats after a restraint stress challenge. SAL+SAL, saline injection followed by another saline injection 1 h later; LPS+SAL, LPS injection followed by a saline injection 1 h later; LPS+Zn, LPS injection followed by a zinc injection 1 h later; SAL+Zn, saline injection followed by a zinc injection 1 h later (n = 10 per group). The data are expressed as the mean ± SEM. (DOCX) [file pone.0120263.s001.docx]

**Zinc prevents sickness behavior induced by lipopolysaccharides after a stress challenge in rats**

**S1 Table. Ultrasonic vocalizations.** Effects of LPS (100 µg/kg) and zinc (ZnSO_4_; 2 mg/kg) on the 22-kHz ultrasonic vocalizations in adult male rats after a restraint stress challenge. SAL+SAL, saline injection followed by another saline injection 1 h later; LPS+SAL, LPS injection followed by a saline injection 1 h later; LPS+Zn, LPS injection followed by a zinc injection 1 h later; SAL+Zn, saline injection followed by a zinc injection 1 h later (*n* = 10 per group). The data are expressed as the mean ± SEM.

|  | **Groups** | | | |  |
| --- | --- | --- | --- | --- | --- |
| Parameters | **SAL+SAL** | **LPS+SAL** | **LPS+Zn** | **SAL+Zn** | *p* |
| Total vocalizations (s) | 15.23±4.99 | 11.15±5.73 | 13.68±5.93 | 20.33±8.67 | 0.7849 |
| Mean vocalizations (s) | 0.10±0.018 | 0.11±0.028 | 0.10±0.022 | 0.09±0.010 | 0.9136 |
| Maximal vocalizations (s) | 0.44±0.11 | 0.38±0.10 | 0.43±0.12 | 0.66±0.28 | 0.6623 |
| Minimal vocalizations (s) | 0.03±0.003 | 0.03±0.001 | 0.03±0.001 | 0.03±0.000 | 0.2931 |
| Total silence (s) | 284.77±4.99 | 288.85±5.73 | 286.32±5.93 | 279.67±8.67 | 0.7849 |
| Mean silence (s) | 34.28±29.59 | 11.32±4.01 | 6.33±2.23 | 3.11±0.78 | 0.4581 |
| Maximal silence (s) | 89.55±26.47 | 121.00±21.75 | 98.43±24.38 | 73.86±17.54 | 0.5319 |
| Minimal silence (s) | 0.05±0.01 | 0.11±0.04 | 0.04±0.001 | 0.05±0.003 | 0.1566 |

One-way ANOVA followed by the Tukey test
